# Karman Vortex Creation Using Cylinder for Flutter Energy Harvester Device

**DOI:** 10.3390/mi8070227

**Published:** 2017-07-21

**Authors:** Ahmed B. Atrah, Mohd Syuhaimi Ab-Rahman, Hanim Salleh, Mohd Zaki Nuawi, Mohd Jailani Mohd Nor, Nordin Bin Jamaludin

**Affiliations:** 1 Department of Mechanical and Materials Engineering, Faculty of Engineering and Built Environment, Universiti Kebangsaan Malaysia (UKM), 43600 UKM Bangi, Selangor, Malaysia; mzn@ukm.edu.my (M.Z.N.); jailani@ukm.edu.my (M.J.M.N.); nurdin@ukm.edu.my (N.B.J.); 2 Directorate General of Electrical Transmission Projects (ETP), Ministry of Electricity, Baghdad 10001, Iraq; 3 Department of Electrical, Electronic, and Systems Engineering, Faculty of Engineering and Built Environment, Universiti Kebangsaan Malaysia (UKM), 43600 UKM Bangi, Selangor, Malaysia; syuhaimi@ukm.edu.my; 4 Institute of Sustainable Energy, Universiti Tenaga Nasional (UNITEN), Jalan IKRAM-UNITEN, 43000 Kajang, Selangor, Malaysia; hanim@uniten.edu.my

**Keywords:** aerodynamic flutter, airflow energy harvesting, flow over a bluff body, Karman vortex, wind energy

## Abstract

This study presents the creation of a Karman vortex for a fluttering electromagnetic energy harvester device using a cylinder. The effects of two parameters, which are the diameter and the position of the cylinder, were investigated on the Karman vortex profile and the amplitude of the fluttering belt, respectively. A simulation was conducted to determine the effect of the creation of the Karman vortex, and an experiment was performed to identify influence of the position of the cylinder on the fluttering belt amplitude. The results demonstrated that vortex-induced vibration occurred at the frequency of the first natural mode for the belt at 3 cm and 10 cm for the diameter and position of the cylinder, respectively. Under such configuration, an electromagnetic energy harvester was attached and vibrated via the fluttering belt inside the turbulent boundary layers. This vibration provides a measured output voltage and can be used in wireless sensors.

## 1. Introduction

The three main techniques for transferring mechanical vibration energy to electric energy are electromagnetic, piezoelectric, and electrostatic techniques. Certain studies have also presented hybrid energy harvesting devices, where two or more transduction mechanisms are utilized for energy harvesting in the same device [1,2,3,4].

The electromagnetic technique can be applied in many scales and produces relatively high power densities given a relatively high velocity between the magnet and coil [5], and smaller internal impedance and larger current output compared to the piezoelectric technique [6,7]. The electromagnetic technique also possesses a good output performance and process compatibility [4], offers a high efficiency energy conversion, and a considerably low frequency operation due to a simple mechanical resonator composition [8]. An electromagnetic generator best suits the frequency up-conversion mechanism harvesting from body-induced motion given its convenience in spring-mass suspension, relatively low resistance, and high power density at the macroscale level [9].

The applications of electromagnetic energy harvesting from structural deformations and vibrations have been intensively developed in the last decade [10,11]. Recent research in energy harvesting by electromagnetic induction has focused primarily on harvesting energy from the mechanical vibration of structures, to which the harvester is attached using the broadband effects [12,13,14]. Some of these researches demonstrated a significant enhancement of the harvested power and the frequency bandwidth of a multimodal vibration energy harvester consisting of arrays of coupled levitated magnets [15,16] when the device is excited beyond its critical Duffing amplitude [17,18]; this is due to magnetic nonlinearity and modal interactions [19].

Recently, scholars have gained considerable interest in renewable energy resources, specifically in wind power. Notably, turbulent flows show various spatial and temporal scales, which provide a unique opportunity for ambient energy harvesting. Voltage can be generated by an electromagnetic energy harvester device that is attached to a belt inside turbulent boundary layers [20]. This output voltage is dependent on the design of the device inside the flow field.

Among micro-wind energy harvesters in the literature, the Windbelt has shown several advantages, such as its small size; its low cost, the fact that it does not require high manufacturing technology or unique materials; is easy to repair and maintain, and; generates higher output power. However, all studies have applied direct airflow without investigating the influence of obstacle bodies inside the airflow tunnel before the belt structure [20,21,22]. In particular, the Windbelt is a new development and still has many areas for improvement. Moreover, the process of manufacturing should be standardized.

One of the suggested devices for energy harvesting in low-speed winds is the Windbelt generator by Frayne and McRae, together with the Humdinger Wind Energy Company [23]. The Windbelt device is a taut belt with high aspect ratio that flutters in low-speed winds [21]. Fei et al. [20] proposed a similar device where the mechanical vibrations are transformed into electrical power via an electromagnetic transducer. These devices motivated the present study of the pre-flutter characteristics of a belt with high aspect ratio (i.e., a membrane strip) in low subsonic flows.

The present study particularly focused on investigating the influences of the diameter and position of a bluff body (cylinder) on the natural frequency and the amplitude and synchronization region of vortex-induced vibrations (VIV) of energy harvesters. Energy harvesting from VIV has received considerable attention due to its distinct properties, self-limited oscillations, and lock-in or synchronization region. The synchronization occurs when vortex shedding frequency is near the natural frequency of the belt structure.

The use of the Karman vortex in energy harvesting has been reviewed and investigated recently using the piezoelectric transduction technique [24,25]. In the present study, the energy harvesting approach used the fluttering belt behind a cylinder through electromagnetic transduction. This work focused on the influence of size and position of the cylinder in achieving the optimum condition for energy harvesting. Notably, by the change of the distance between the cylinder and the belt and their diameters, the particular wavelength of pressure oscillations could be adjusted to the amplitude of the belt due to the interference effect. The present study aims to explore the effects of cylinder diameter on the creation of the airflow vortex, as well as the effects of the cylinder position on the oscillating frequency and amplitude of the membrane. The use of the Karman vortex in belt fluttering electromagnetic energy harvester design has not been studied in the literature.

One of the important applications of this type of energy harvester device is the wireless sensors in buildings for energy spending monitoring, security surveillance, structural health monitoring, and damage detection.

This paper is divided into six sections. Section 2 discusses the model setup and theoretical background. Section 3 presents the parametric analysis for the creation of the vortex for different diameters of a cylinder. The effects of different positions of the bluff body on the amplitude of the fluttering belt is presented in Section 4. Open-circuit voltage for the optimum parameters is discussed in Section 5. Finally, Section 6 presents some concluding remarks.

## 2. Energy Harvester Device

### 2.1. Model Setup

**Figure 1:**
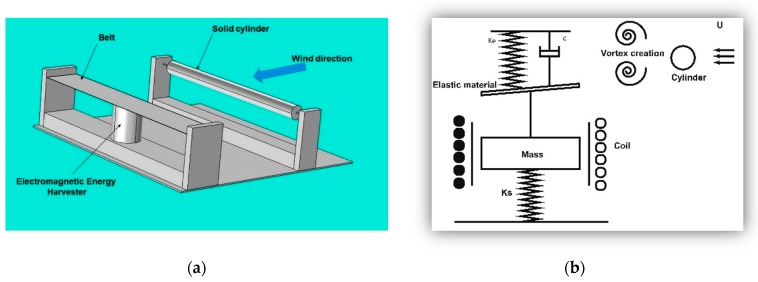
(**a**) Physical model configuration; (**b**) Architecture of the developed energy harvester.

To investigate the influences of the factors mentioned in the introduction on the flutter amplitude of the belt, a test model was simulated, designed, and fabricated as shown in Figure 1.

In energy harvesting, a vortex is generated by placing a cylinder to disturb the fluid flow. The fluid flow pressure exerts a force on the membrane strip, which causes the belt to deform, thereby inducing mechanical stress on the electromagnetic energy harvester. The mechanical vibration causes a charge in the electromagnetic harvester, which is then used by the harvester circuit.

The electromagnetic harvester comprises a wound coil and a cylindrical permanent magnet. When the device is exposed to vibrations, the magnet attached to it starts moving because the coil experiences a changing magnetic flux density; an electromagnetic field (EMF) is induced in the coil according to Faraday’s law of electromagnetic induction. Similarly, when the ambient wind surge flows over the belt, it produces an upward lift force; however, given that the natural wind flow is always fluctuating, the harvester attached to the belt will start oscillating vertically. Consequently, an EMF will be produced in the coil. The EMF induced in the coil depends on the number of coil turns, magnetic field strength, and the relative velocity between the magnet and coil. However, the characterization of the electromagnetic transducer is beyond the scope of this study and is, therefore, the subject of future work of the researchers.

### 2.2. Theoretical Background

The power density *P* in the wind is related to the cube of the wind speed, which can be expressed as:

(1) $$P=\frac{1}{2}\cdot \rho \cdot U^{2}$$

where the *ρ* and U represent air density and wind speed, respectively. The belt, cylinder, and flow medium interactions can be described by the governing equations of each subsystem with the addition of the appropriate coupling terms [20,22,26]. In flow speeds, less than 0.3 of the sound velocity is assumed to be incompressible flow. A Newtonian fluid, such as air, can be described by the continuity equation:

(2) $$\frac{D\rho }{Dt}+\rho S_{kk}=0$$

and Navier–Stokes:

(3) $$\rho \frac{Dv_{i}}{Dt}=-\frac{\partial p}{\partial x_{i}}+\frac{\partial \tau_{ij}}{x_{j}}$$

VIV is an expression that studies the vibration of the object because of the vortices generated from flowing fluid. The maximum fluctuation of the belt can occur when the natural frequency of the belt is near to the shedding frequency of the vortex [27]. The Strouhal number (*S_t_*) is a dimensionless number, which describes the mechanism of the oscillating flow and can be expressed as:

(4) $$S_{t}=\frac{f_{s}D}{U}$$

where $f_{s}$ is the vortex shedding frequency of a body at rest (i.e., Strouhal frequency),  D is the characteristic length, and U is the velocity of the ambient flow.

Flutter is a well-known dynamic excitation phenomenon in wind engineering (e.g., of bridges), where a structure becomes aerodynamically unstable through a coupled motion in the vertical bending and the torsional direction. The simplest form of the flutter motion has two degrees of freedom and this form similar to a second-order system which can be expressed as follows [20]:

(5) $$m\ddot{h}+2m\zeta_{h}\omega_{h}\dot{h}+m\omega_{h}^{2}h=F_{L}\left(t\right)$$

(6) $$M_{\alpha }\ddot{\alpha }+2M_{\alpha }\zeta_{\alpha }\omega_{\alpha }\dot{\alpha }+M_{\alpha }\omega_{\alpha }^{2}\alpha =F_{M}\left(t\right)$$

where m and $M_{\alpha }$ are the mass per unit length and the moment of inertia (rotational mass), respectively; $\zeta_{h}$ and $\zeta_{\alpha }$ are the damping ratios in bending and torsional modes, respectively; h and α represent deflection and rotation, respectively; and $\omega_{h}$ and $\omega_{\alpha }$ are the natural circular frequencies for the bending and torsional modes, respectively. Equations (5) and (6) illustrate the aerodynamic forces contributing to flutter of belt, which are a vertical lift force, *F_L_(t)*, as well as a pitching moment, *F_M_(t)*, induced by the interaction between airflow, *U*, and oscillations in the structure.

The motion of the flutter-based energy harvester section can be described as a forced vibration equation, as shown as follows [28]:

(7) $$m\ddot{z}\left(t\right)+c_{t}\dot{z}\left(t\right)+kz\left(t\right)=F_{wind}\left(t\right)$$

where m, $c_{t}$, and *k* are the mass, total damping and stiffness of the vibration frame, respectively; z denotes the relative displacement between the magnet and coil; and $F_{wind}\left(t\right)$ is the motion-induced force that corresponds to wind flow. The total damping coefficient (of the harvester) $c_{t}$ includes the mechanical and electrical damping (i.e., $c_{t}=c_{m}+c_{e}$). The mechanical damping ($c_{m}=2m\zeta_{m}\omega_{n}$) is expressed in terms of the mass *m*, mechanical damping ratio $\zeta_{m}$, and the natural frequency, $\omega_{n}$. The electrical damping $c_{e}$ is expressed as [29]:

(8) $$c_{e}=\frac{{\left(Nl\beta \right)}^{2}}{R_{L}+R_{c}+j\omega L_{c}}$$

where N, l, and β are the number of coil turns, the coil length exposed to the magnetic flux, and the average flux density, respectively; $R_{L}$, $R_{c}$, and $L_{c}$ are the load resistance, coil resistance, and coil inductance, respectively; and ω is the angular frequency in the motion between the magnet and coil. The coil inductance is generally negligible.

The generated energy is equivalent to the energy extracted by the electrical damping from the system, as shown as follows [28]:

(9) $$P=c_{e}{\dot{z}}^{2}$$

where $\dot{z}$ is the relative speed between the magnet and coil; it is a function of electrical damping, $c_{e}$, given that the total damping, $c_{t}$, including $c_{e}$, suppresses displacement *z.* That is, power generation depends on the electrical and structural parameters.

## 3. Parametric Analysis for Vortex Creation

**Figure 2:**
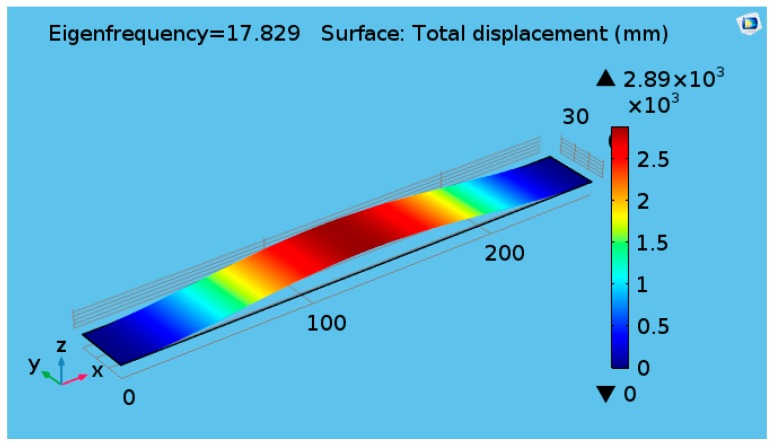
First mode response of the belt.

In this section, only numerical simulations are performed to investigate the effects of cylinder diameter on the vortex creation behind the cylinder. The belt was designed to have a resonance near to the shedding frequency of the vortex. The response of the belt has a peak of 17.829 Hz, which is close to the computed resonant frequency of the belt at 15.5 Hz (from a separate eigenfrequency analysis of the strap), as shown in Figure 2.

The Strouhal number is assumed to be constant for a broad range of Reynolds numbers and is approximated as 0.2 for cylindrical bluff bodies [30] and 0.15 for flat plate [27]. In this study, the fluttering belt is assumed as a flat plate. For the airflow velocity of 3 m/s and characteristic length of 25 mm, the vortex shedding frequency was found to be 18 Hz; this result is near to the first mode of the natural frequency.

**Figure 3:**
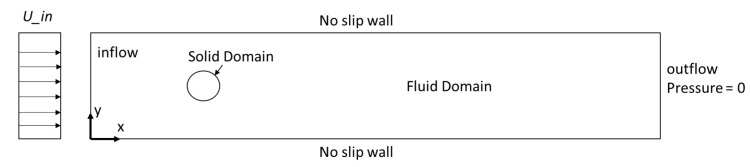
Domain and boundary conditions for vortex analysis.

Figure 3 illustrates the following numerical problem domains associated with 2D flutter: (i) the fluid domain occupied by incompressible flows; (ii) the structural domain; and (iii) the interface region, including inlet and outlet.

The dependent variables for this solution domain include pressure p and the fluid particle velocity vector **u** = {*u*, *v*, *w*}^T^, where the *u*, *v*, and *w* are the components of velocity, *x*, *y*, and *z* are the Cartesian directions. To simplify the problem, the fluid flow is assumed to be laminar and Newtonian, and the constant air density ρ is 1 kg/m^3^. In this study, 2D simulations were conducted using COMSOL Multiphysics (COMSOL Inc., Stockholm, Sweden) to predict the Karman vortex creation behind the solid cylinder during flow.

**Figure 4:**
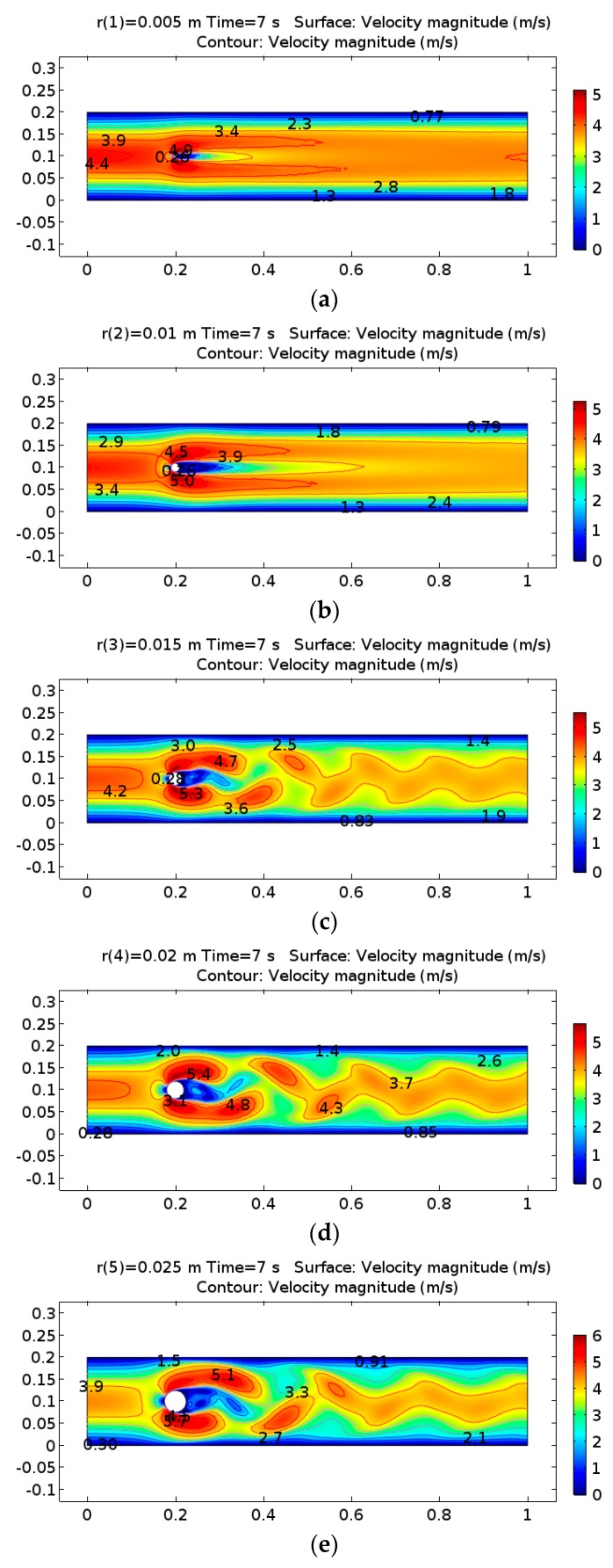
Development of fluid velocity contour demonstrating von Karman vortex streets behind a bluff body. (**a**) Diameter = 1 cm; (**b**) Diameter = 2 cm; (**c**) Diameter = 3 cm; (**d**) Diameter = 4 cm; (**e**) Diameter = 5 cm.

The diameter of the cylinder was set using a parametric sweep function from 1 cm to 5 cm with 1 cm steps. When the airflow speed was 3 m/s and the diameter was 1 cm, the laminar flow was lightly disturbed while passing through the cylinder. Figure 4a,b show the velocity field behind the solid cylinder. When the cylinder diameter was set to 3 cm, a von Karman vortex street appears with a predictable frequency and involves the shedding of eddies from alternating sides (Figure 4c–e). The parametric sweep function was added to find the solution to a sequence of time-dependent problems that arise when some parameters of interest are varied. With a time-dependent study, PARDISO Solver, which is a Jacobian update, was selected to operate once per time step to compute a new Jacobian on the first iteration of each time step. Mesh sets are a free triangle node and consist of 5904 domain elements and 354 boundary elements.

## 4. Experimental Results and Discussion

**Figure 5:**
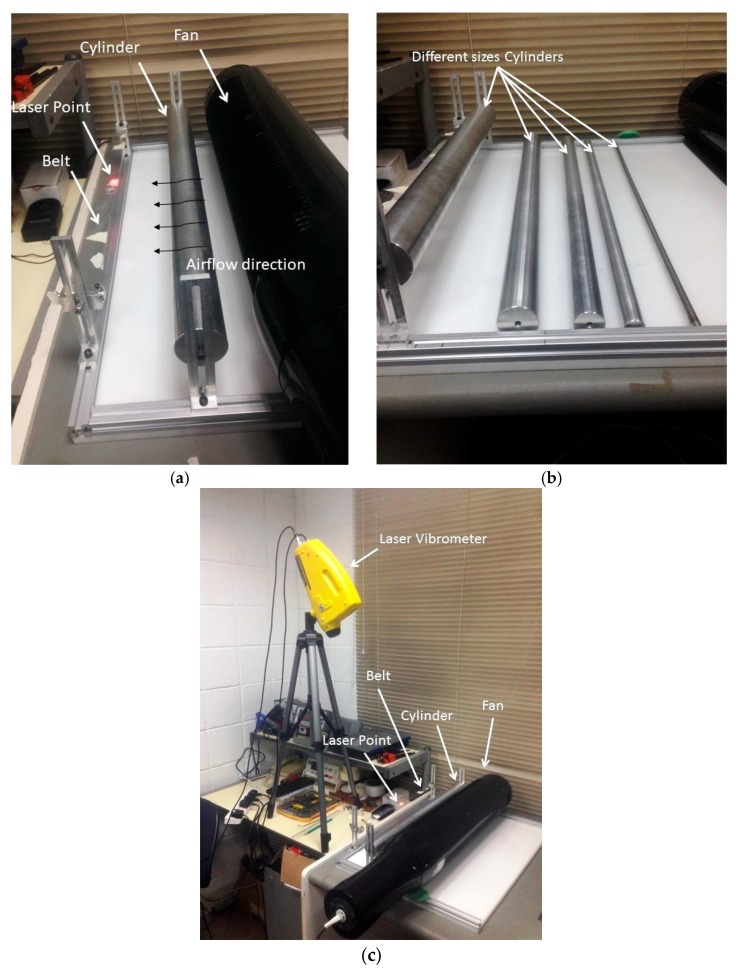
(**a**) Measurement point at the center of the belt and airflow direction. (**b**) Different sizes of cylinders. (**c**) Laser Vibrometer.

This section focuses on the interference between flow disturbing conditions by using the cylindrical bluff bodies of differing radii with the various distances in the configuration shown in Figure 5b.

The distance between the belt and wind source is 16.5 cm, and the belt size is 50 cm × 3 cm × 0.175 mm (Figure 5). This experiment aims to investigate the maximum amplitude of the fluttering belt due to disturbed airflow by the cylinder to generate a vortex in the region before the belt. The air speed was generated by using a fan in a horizontal direction with a fixed speed of 3 m/s, the average testing time was 60 s, and the measurement point was taken at the center of the belt.

A laser vibrometer and acquisition data (PULSE) were used in measuring the vertical displacement of the belt to analyze the vibration amplitude of the fluttering belt. Five aluminum cylinders, which have diameters ranging from 1 cm to 5 cm, were utilized in this experiment, and the distances swept were from 2.5 cm to 15 cm with 2.5 cm steps.

**Figure 6:**
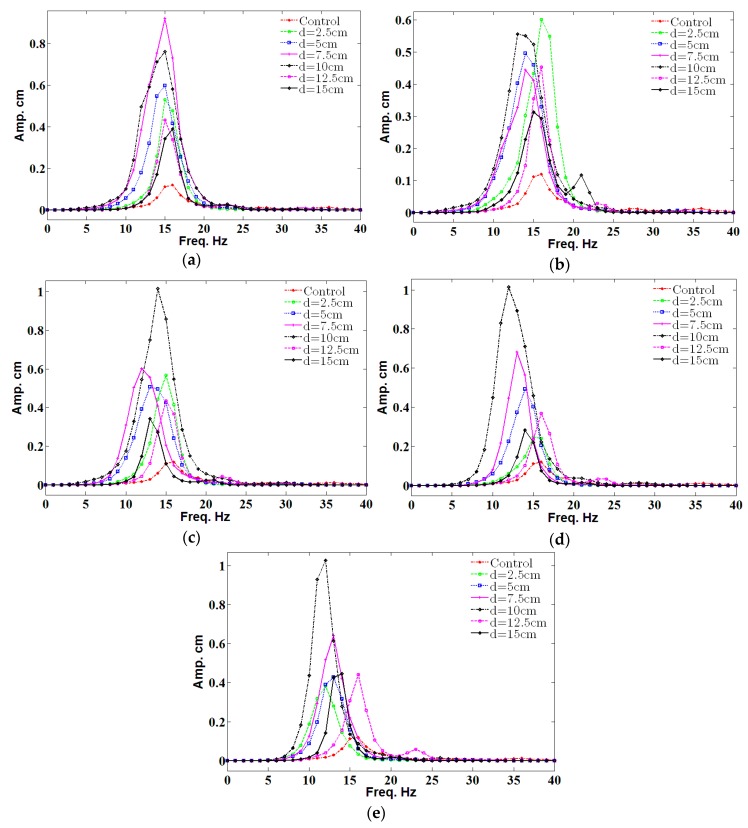
The fluttering amplitude for different diameters of the cylinder. (**a**) Diameter = 1 cm; (**b**) Diameter = 2 cm; (**c**) Diameter = 3 cm; (**d**) Diameter = 4 cm; (**e**) Diameter = 5 cm.

In Figure 6, the maximum amplitude, which represents the resonance, of the fluttering belt without a cylinder (control) has the minimum value for all distances between the cylinder and belt compared with all the diameters of the cylinder. The maximum amplitude of almost 1 cm occurred for the cylinder diameters of 3, 4, and 5 cm with a distance of 10 cm, as shown in Figure 6c–e, respectively. However, the fluttering frequency was minimal in a large diameter.

**Figure 7:**
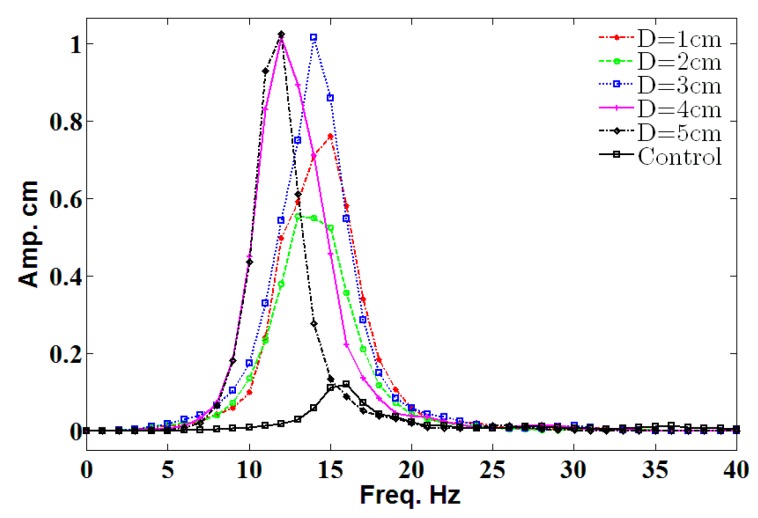
The amplitude value for different diameters in a distance of 10 cm.

Figure 7 shows the amplitude value for different cylinder diameters (*D*) in a frequency range of 0 Hz to 40 Hz with a position of 10 cm. Finding shows that 1 cm of amplitude was recorded for *D* = 3 cm, *D* = 4 cm, and *D* = 5 cm. Moreover, the maximum value only occurred at the resonance; this result agrees with the literature. The optimum diameter for the cylinder is 3 cm because it is near to the resonant frequency of the belt.

## 5. Open-Circuit Voltage

**Figure 8:**
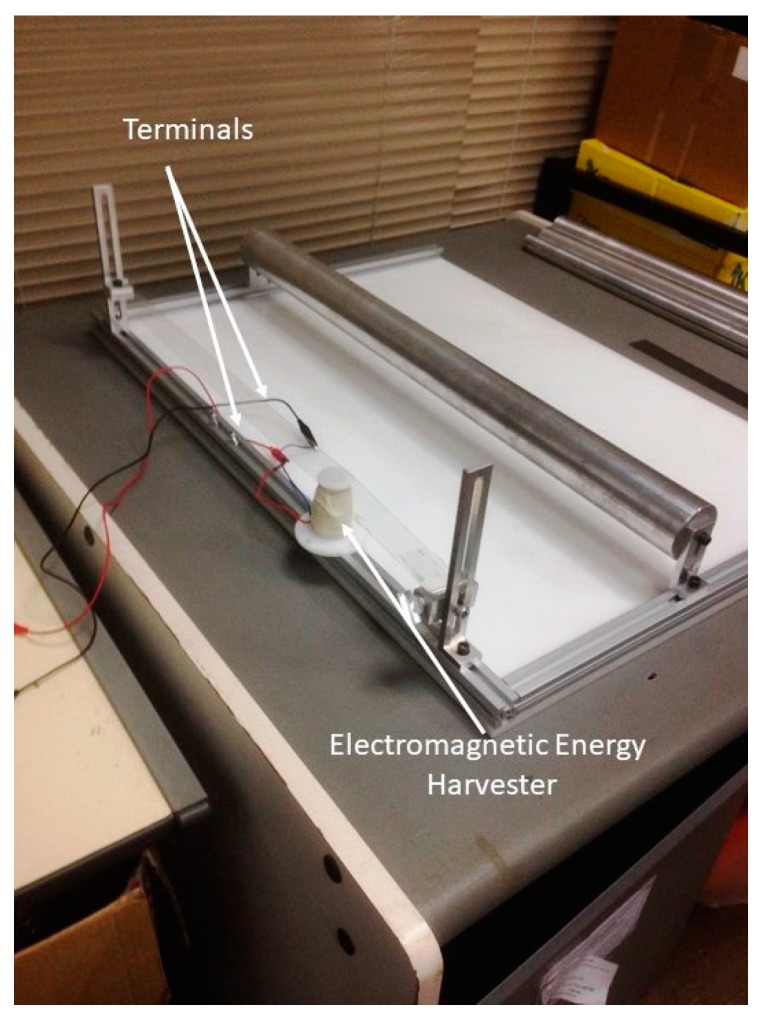
The full experimental system of an energy harvester.

**Figure 9:**
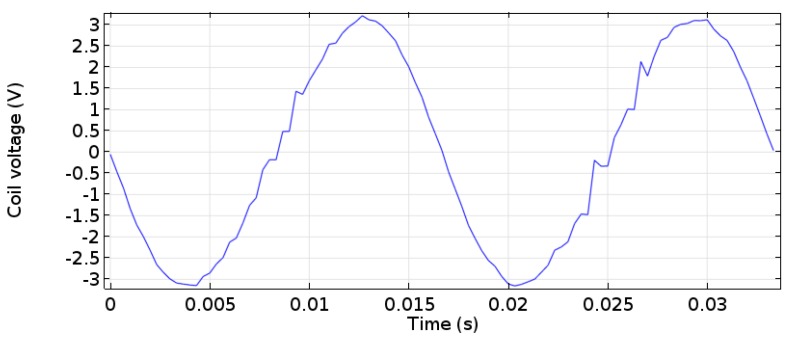
Measured open-circuit voltage waveform of the Windbelt flutter energy harvester device.

From the simulations and experiments, the optimum parameters were used with the electromagnetic energy harvester placed near the long end to harvest the energy. The parameters are 10 cm for the cylinder distance from the belt, which provides the belt a high amplitude, and 3 cm for the diameter of the cylinder, which is near to the shedding frequency (Figure 8). In this work, only the open-circuit voltage was measured, and no resistive circuit or load was applied given that the objective of this work is to investigate the feasibility of vortex creation and flutter in harvesting energy. The result for the coil voltage peak-to-peak was 6 V, as shown in Figure 9 for 5000 turns of the copper coil and neodymium (NdFeB) cylindrical magnets.

## 6. Conclusions

A novel energy harvester from wind energy is proposed in this study. This energy harvester allows the creation of a Karman vortex behind a cylinder to induce vibrations for the fluttering belt of an electromagnetic energy harvester. A simulation study of the vortex creation was performed to investigate the effects of cylinder diameter on the vortex profile. An experimental study was conducted to determine the effects of the cylinder position on the amplitude of the belt. The optimum values of the diameter and position of cylinder were found to be 3 cm and 10 cm, respectively. These results match the resonance frequency of the belt of the energy harvester, which provides the maximum enegry harvesting. A 6-V peak-to-peak open-circuit voltage was measured for the coil of the electromagnetic energy harvester. Future work would be conducted on the resistive circuit to investigate the optimum load for this energy harvester device.
